# Epidemiology of mucopolysaccharidoses (MPS) in United States: challenges and opportunities

**DOI:** 10.1186/s13023-021-01880-8

**Published:** 2021-05-29

**Authors:** Yana Puckett, Alejandra Mallorga-Hernández, Adriana M. Montaño

**Affiliations:** 1 Department of Epidemiology, Saint Louis University College for Public Health and Social Justice, 3545 Lafayette Avenue, St. Louis, MO 63104 USA; 2 Saint Louis University School of Medicine, St. Louis, MO USA; 3 Department of Pediatrics, Edward A. Doisy Research Center, Saint Louis University School of Medicine, 1100 South Grand Blvd., Room 313, St. Louis, MO 63104 USA; 4 Department of Biochemistry and Molecular Biology, Edward A. Doisy Research Center, Saint Louis University School of Medicine, St. Louis, MO USA

**Keywords:** Incidence, Prevalence, Newborn screening, Rare disease registry

## Abstract

**Background:**

Mucopolysaccharidoses (MPS) are rare, inherited lysosomal storage disorders characterized by progressive multiorgan involvement. Previous studies on incidence and prevalence of MPS mainly focused on countries other than the United States (US), showing considerable variation by country. This study aimed to identify MPS incidence and prevalence in the US at a national and state level to guide clinicians and policy makers.

**Methods:**

This retrospective study examined all diagnosed cases of MPS from 1995 to 2015 in the US using the National MPS Society database records. Data included year of birth, patient geographic location, and MPS variant type. US population information was obtained from the National Center for Health Statistics. The incidence and prevalence rates were calculated for each disease. Incidence rates were calculated for each state.

**Results:**

We obtained information from 789 MPS patients during a 20-year period. Incidence of MPS in the US was found to be 0.98 per 100,000 live births. Prevalence was found to be 2.67 per 1 million. MPS I, II, and III had the highest incidence rate at birth (0.26/100,000) and prevalence rates of 0.70–0.71 per million. Birth incidences of MPS IV, VI, and VII were 0.14, 0.04 and 0.027 per 100,000 live births.

**Conclusions:**

This is the most comprehensive review of MPS incidence and prevalence rates in the US. Due to the large US population and state fragmentation, US incidence and prevalence were found to be lower than other countries. Nonetheless, state-level studies in the US supported these figures. Efforts should be focused in the establishment of a national rare disease registry with mandated reporting from every state as well as newborn screening of MPS.

## Background

Mucopolysaccharidoses (MPS) are rare and heterogeneous, inherited lysosomal storage disorders characterized by progressive multiorgan involvement. MPS are caused by defects in genes coding for different lysosomal enzymes which degrade glycosaminoglycans (GAGs) such as heparan sulfate (HS), chondroitin sulfate (CS), dermatan sulfate (DS) and keratan sulfate (KS). The deficient enzyme activity results in systemic storage of GAGs and a wide array of clinical manifestations. There are 11 known enzyme deficiencies and seven forms of MPS, some of which have different subtypes.

MPS I patients have deficiency of α-l-iduronidase enzyme (*IDUA*; MIM 252,800), which degrades DS and HS. Depending on the severity of the clinical presentation, MPS I can be manifested in three different phenotypes: Hurler, Hurler–Scheie or Scheie syndromes [1]. MPS II, also known as Hunter syndrome, is an X-linked recessive disorder characterized by the deficiency of iduronate-2-sulfatase (*I2S*; MIM 309,900), which similarly to *IDUA* also degrades DS and HS. MPS II patients have mild or severe clinical presentation, and although it can be similar to MPS I in its severe form, MPS II shows moderate central nervous system progression [1, 2]. MPS III (Sanfilippo syndrome) presents four subtypes: MPS IIIA-D, in which HS is not degraded. The aforementioned presentations are a result of the limited enzymatic activity of heparan-*N*-sulfatase (*SGSH*; MIM 252,900), α-*N*-acetylglucosaminidase (*NAGLU;* MIM 252,920), α-glucosaminidase acetyltransferase (*HGSNAT*; MIM 252,930), or *N*-acetylglucosamine 6-sulfatase (*GNS*; MIM 252,940), respectively [3–6]. MPS IV, also referred to as Morquio syndrome, presents two subtypes: MPS IVA and MPS IVB, caused by the lack of *N*-acetylgalactosamine-6-sulfate sulfatase (*GALNS;* MIM 253,000) and β-galactosidase (*GLB1;* MIM 253,010) respectively. MPS IVA is characterized by accumulation of two GAGs: KS and C6S, while MPS IVB results in accumulation of KS solely [7, 8]. MPS VI (Maroteaux-Lamy syndrome) results from deficiency of *N*-acetylgalactosamine-4-sulfatase (*ARSB;* MIM 253,200), which causes accumulation of DS and C4S [9]. MPS VII, also known as Sly syndrome, causes accumulation of DS, C4S, C6S, and HS due to inactivity of β-d-glucuronidase (*GUS;* MIM 611,499). MPS VII is the most rare and lethal MPS. It can present with hydrops fetalis, which results in high newborn mortality [10, 11]. Finally, MPS IX, which results from mutations in the *HYAL1* gene, is characterized by markedly elevated plasma hyaluronan concentration, and the complete deficiency of plasma hyaluronidase activity [12]. This MPS type, has only been described in four patients since 1996 in the US [12, 13], and no further cases have been noted in international MPS studies [14]. Although the incidence of all seven types of MPS presents a wide range and varies geographically, their collective incidence has been reported to be approximately 1 per 25,000 [15].

Several MPS can be treated with enzyme replacement (ERT) or hematopoietic stem cell transplantation. Nonetheless, success of these interventions heavily relies on early detection. Early treatment can improve outcomes and prevent long-term deterioration due to progressive accumulation of GAGs.

Although many European and Asian countries have reported the incidence and prevalence of MPS disorders [16], its epidemiology has not been studied at a national level in the United States (US). In the last decade, newborn screening pilot studies in various US states have provided insight into the regional incidence of MPS I. However, precise incidence and prevalence data on all MPS types in the US is quite limited. These data are necessary to address the challenge of securing public health funding for treatment and for supporting fundamental biomedical research [17–19].

The current lack of epidemiological data on rare lysosomal diseases also affects whether states test for them in newborns [20]. Data are needed to determine which is the earliest, cost-effective, and least invasive way of screening for a disease. Early detection and intervention of some of the rare diseases lowers morbidity and improves outcomes, including better quality and length of life [21–24]. In the US, the expansion of the newborn screening panel depends on the Advisory Committee on Heritable Disorders in Newborns and Children (ACHDNC). They in turn, advise the US Secretary of Health on including specific conditions in the Recommended Universal Screening Panel. This key recommendation is based on: (1) availability of disease screening and treatment, (2) net benefits of screening to usual clinical care, (3) state laws, (4) cost and funding sources, (5) frequency of the disorder in the state, and (6) readiness of public health departments to implement population screening [25, 26]. Currently in the US only MPS I meets this inclusion criteria. The most prevalent methodological approach to support the inclusion of a condition to the newborn screening panel consists of creating a model that estimates outcomes for identical cohorts of newborns identified to have the rare condition via clinical identification versus identification through newborn screening. The determination of prevalence and incidence of MPS in the US will provide the number of cases diagnosed via clinical identification, contributing to the elaboration of the models necessary to advance newborn screening.

The lack of epidemiological data on MPS types in the US deprives us of evidence to support seeking funds to expand newborn screening or further research in this field [25]. Currently, only 17 of 50 US states screen for MPS. These screenings include only MPS I, one of the most common MPS [27]. Most of these states have adopted MPS I in their newborn screening panel only in the last year, with the most recent being Vermont (May 2019) [28]. The objective of this study is to determine the prevalence and incidence of all MPS in the US at a national and state level using the national MPS society registry as our main source, and MPS VII data from Ultragenyx Pharmaceuticals.

## Results

### Incidence of MPS in the US

Between 1995 and 2015, 721 MPS patients registered with the National MPS Society. 681 MPS patients were classified appropriately according to their MPS type. Ultragenyx reported that as of 2015, 21 MPS VII patients were identified and enlisted in their registry program in the US. Additionally, the International Registry for MPS IVA had a total of 87 patients in the US between 1998 and 2006 [7].

The average total population in the US in the same study period was 295,130,476 and the overall population prevalence was found to be 2.67 per million. Moreover, the total number of live births in the US over these two decades was 80,118,336. The calculated incidence rates based on these data are shown in Table 1.

**Table 1:** Overall incidence and prevalence rates of MPS in United States (1995–2015) based on the National MPS Society membership

MPS type	Number of patients	Percentage of all MPS (%)	Incidence per 100,000	Prevalence per 1,000,000
MPS I (Hurler Syndrome)	207	26.23	0.26	0.70
MPS II (Hunter Syndrome)	207	26.23	0.26	0.70
MPS III (Sanfilippo Syndrome)	210	26.60	0.26	0.71
Type A	153	19.40	0.19	0.52
Type B	40	5.10	0.05	0.14
Type C	12	1.50	0.015	0.04
Type D	0	0.00	0	0
MPS IV (Morquio Syndrome)	113	14.32	0.14	0.38
Type A^a^	87	11.00	0.11	0.29
Type B	3	0.40	0.004	0.01
MPS VI (Maroteaux-Lamy Syndrome)	31	3.92	0.04	0.11
MPS VII^b^ (Sly Syndrome)	21	2.70	0.027	0.07
MPS IX (Hyaluronidase deficiency)	0	0.00	0	0
All Mucopolysaccharidoses	789	100.00	0.98	2.67

Some patients are reported as MPS III or IV while there is no information on the sub-type

^a^Based on information from the MPS IVA International registry [7]

^b^Based on information provided by Ultragenyx Pharmaceuticals

**Fig. 1:**
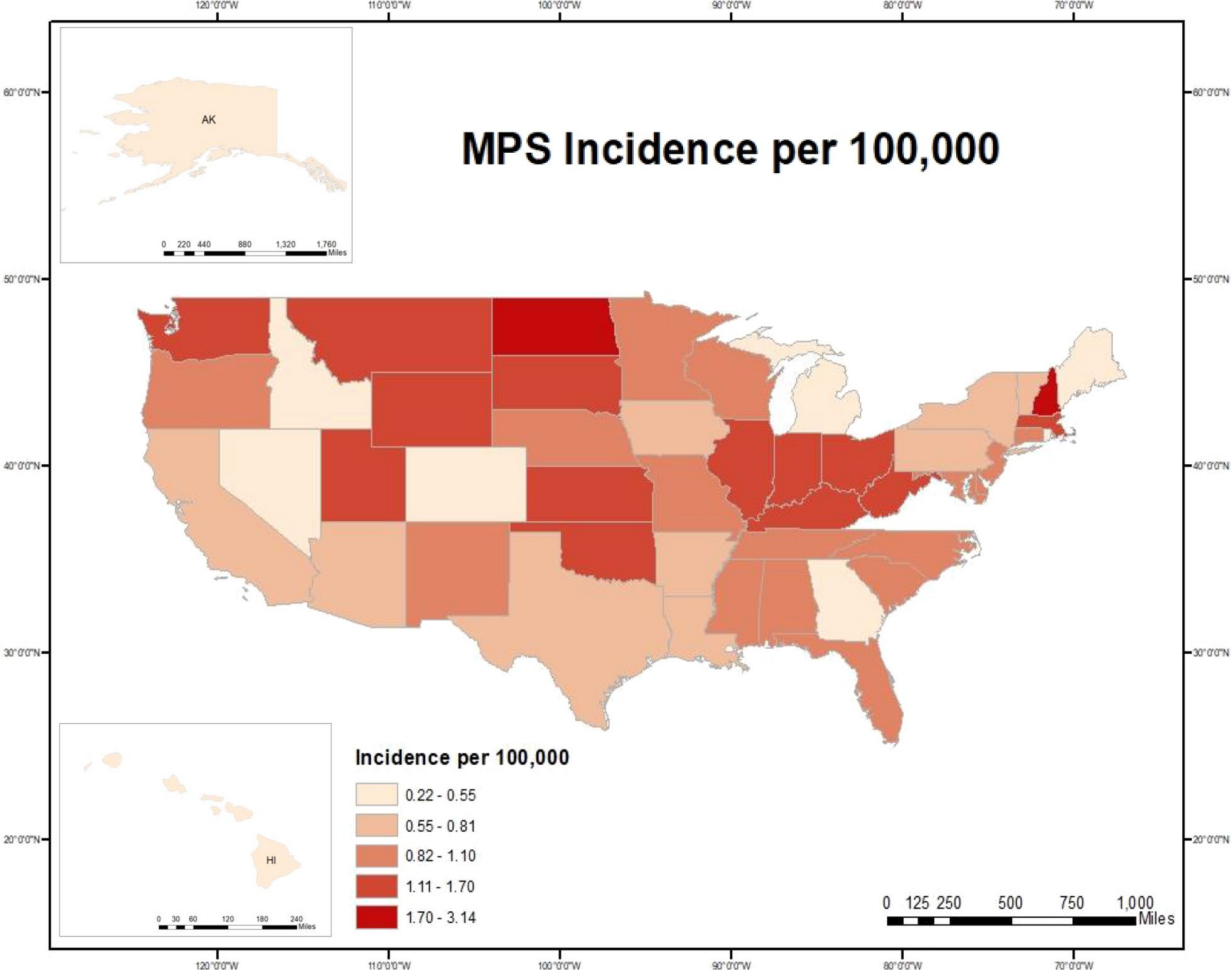
Incidence of MPS in the United States

The combined incidence for all MPS was 0.98 per 100,000 live births. MPS I, II and III had the highest incidence of all types: 0.26 per 100,000 live births each. MPS IV, VI, and VII had incidences of 0.14, 0.04 and 0.027 per 100,000 live births. No cases of MPS IX were reported in the National MPS Society. MPS incidence was analyzed per state (Table 2), then plotted on a heat map (Fig. 1). States with the highest incidence were New Hampshire (3.14), North Dakota (2.46), Massachusetts (1.71), Montana (1.62) and Utah (1.55). States with the lowest MPS incidence included Idaho (0.22), Hawaii (0.26), Maine (0.39), Rhode Island (0.39), and Alaska (0.44).

**Table 2:** Incidence rates of MPS by state in the United States per 100,000 live births (1995–2015) based on the National MPS Society membership

State	Number of patients	Number live births total	Incidence per 100,000
Alabama	12	1,191,832	1.01
Alaska	1	227,511	0.44
Arizona	13	1,724,510	0.75
Arkansas	6	818,434	0.73
California	77	10,160,112	0.76
Colorado	7	1,324,305	0.53
Connecticut	7	743,272	0.94
Delaware	2	222,737	0.90
Washington DC	1	184,676	0.54
Florida	38	4,383,704	0.87
Georgia	15	2,662,971	0.56
Hawaii	1	379,247	0.26
Idaho	1	458,475	0.22
Illinois	38	3,237,954	1.17
Indiana	22	1,687,025	1.30
Iowa	6	786,264	0.76
Kansas	11	792,446	1.39
Kentucky	16	1,125,145	1.42
Louisiana	9	1,271,230	0.71
Maine	1	259,668	0.39
Maryland	14	1,466,742	0.95
Massachusetts	25	1,459,220	1.71
Michigan	13	2,308,863	0.56
Minnesota	14	1,396,608	1.00
Mississippi	7	801,068	0.87
Missouri	17	1,530,183	1.11
Montana	4	247,212	1.62
Nebraska	5	525,692	0.95
Nevada	4	721,957	0.55
New Hampshire	8	254,781	3.14
New Jersey	19	2,105,921	0.90
New Mexico	5	543,696	0.92
New York	37	4,811,483	0.77
North Carolina	21	2,429,993	0.86
North Dakota	5	203,408	2.46
Ohio	35	2,817,693	1.24
Oklahoma	15	1,070,197	1.40
Oregon	10	917,976	1.09
Pennsylvania	21	2,857,974	0.73
Rhode Island	1	113,926	0.39
South Carolina	11	1,162,144	0.95
South Dakota	3	243,296	1.23
Tennessee	14	1,621,494	0.86
Texas	48	7,825,179	0.61
Utah	16	1,035,564	1.55
Vermont	1	121,549	0.82
Virginia	23	2,067,341	1.11
Washington	21	1,746,394	1.20
West Virginia	6	416,892	1.44
Wisconsin	12	1,359,168	0.88
Wyoming	2	153,559	1.30

### Current study incidence by state vs. newborn screening pilot studies for MPS I

To date, 17 US states have included MPS I in their newborn screening panel. Many screening laws resulted from pilot studies proving the feasibility, accuracy, and efficacy of screening newborns for lysosomal disorders, including MPS I [29]. Some states published their pilot study data [29–36], allowing the authors to calculate state-level incidence and compare it with the current study data.

The largest newborn screening pilot studies in the country include Missouri (Total number screened (n) = 308,000) and Illinois (n = 219,793), whose incidence rates were found to be 0.65 and 0.45 per 100,000 live births respectively (Table 3) [29, 31]. The MPS I incidence calculated from the National MPS Society database was 0.46 in Missouri and 0.43 in Illinois. The incidence for Illinois is remarkably similar in both studies; Missouri rates are also comparable, although lower in the current study (Table 4). These prospective pilot studies included all the state’s newborns for more than a year, which allowed an accurate incidence calculation.

**Table 3:** Findings of MPS I newborn screening in six different states

States	Missouri	Illinois	Washington	North Carolina	Kentucky	New York
Screened positive	133	151	9	54	57	13
True positive	2	1	3	1	1	0
Carriers	8	5	1	2	NA	4
Pseudodeficiency	71	30	0	13	NA	8
False-positive results	45	87	5	NA	NA	0
Undetermined disease status	2	4	NA	3	NA	1
Unresolved/status pending	5	24	NA	NA	NA	0
Lost/refused follow-up	0	0	NA	1	NA	0
Total number of screened newborns	308,000	219,793	106,526	62,734	55,161	35,816
Incidence per 100,000	0.65	0.45	2.81	1.59	1.81	0

**Table 4:** Comparison of incidence rates of MPS I between the present study and newborn screening pilot studies

States	Incidence rate of present study	Pilot studies incidence rates
Missouri	0.46	0.65
Illinois	0.43	0.45
Washington	0.34	2.81
North Carolina	0.29	1.59
Kentucky	0.98	1.81
New York	0.19	0

However, in several states that conducted smaller newborn screening pilot studies, there was more divergence between those pilot results for MPS I and results of the current study. Washington (n = 106,526), North Carolina (n = 62,734), Kentucky (n = 55,161) and New York (n = 35,816), conducted smaller newborn screening pilot studies than Missouri and Illinois, which yielded higher incidence rates of MPS I than our study (Tables 3, 4) [30, 32, 33, 36]. It is possible that these figures diverge because of the smaller sample size of these pilot studies or to the high incidence of pseudodeficient alleles in the normal population leading to false positive results [37].

In addition to the MPS I newborn screening pilot studies that allowed the authors to compare MPS I incidence accurately in the US, the literature was reviewed to identify state-level incidence for all the other MPS types. For MPS II, the Hunter Outcome Survey was used to estimate incidence in the US for comparison. The Hunter survey is a global, multi-center, long-term, observational survey overseen by national, regional, and global scientific advisory boards [38]. It reported a total of 45 MPS II patients in North America as of 2007. The calculated incidence using the Hunter survey between 1995 and 2007 would be 0.11 per 100,000. However, the incidence rate found in our study is higher (0.26 per 100,000). Thus, an estimated incidence rate in the US is somewhere between 0.11 and 0.26 per 100,000.

For MPS IVA, the International Registry for Morquio A Disease showed a total of 87 patients in the US between 1998 and 2006 [7]. The total number of live births in the US between 1998 and 2006 was 36,313,349. This calculates to an incidence of 0.24 per 100,000. This was higher than our calculated incidence for MPS IVA from the National MPS Society, at 0.14 per 100,000. Thus, incidence of MPS IVA in the US is in the range of 0.14–0.24 per 100,000. Information was not available for MPS IVB, and no reporting publication on MPS IVB incidence rate in the U.S was found.

No publications reporting MPS III or MPS VI incidence rates in the US were found. For MPS VII, a patient registry thought to be complete for the US was generously provided by Ultragenyx® Pharmaceuticals, the manufacturer of enzyme for MPS VII ERT. Based on their records, the incidence rate for MPS VII in the US is 0.027 per 100,000.

## Discussion

### National versus global incidence

From 1995 to 2015, the MPS incidence in the US was found to be 0.98 per 100,000 live births. Compared to other nations, this figure falls on the lower spectrum. Most rates range from 1.53 to 4.8 per 100,000 live births for combined MPS [39, 40].

This variability in incidences by nation may reflect the properties of the Mendelian genetic inheritance of the disease. Countries with enclosed populations and little migration in and out to increase genetic diversity are likely to have higher incidence. Consanguinity and founder effect may be at work in these populations. Consanguinity marriages are associated with higher frequency of autosomal recessive disorders, more precisely between first cousins [41]. A study conducted in Tunisia examined 14 families with MPS I and examined consanguinity associations using molecular analysis and pedigree construction, concluding that consanguineous marriages play a role in the high frequency of autosomal recessive disorders in Tunisia due to its incidence increase of certain molecular mutations leading to MPS I [41].

Additionally, the highest incidence of MPS I was 3.8 cases per 100,000 live births in Northern Ireland. Northern Ireland has a high proportion of Irish traveler population that tends to intermarry [42]. Furthermore, a 2010 study found Saudi Arabia to have some of the highest MPS rates, and a combined incidence of 16.9 shows the effect of consanguinity [43]. Likewise, some communities in Northern Brazil and Eastern Europe have illustrated founder effect for MPS VI [44, 45]. In addition, MPS I, III and IVA founder effect has been observed in the Middle East, Sweden, Cayman Islands, and Japan [46–49].

For MPS I, the national incidence ranged from 0.11 to 100,000 in Taiwan [50] to 1.85 per 100,000 in Norway [51]. In this study, the US incidence was found to be 0.26, which is included in this range, similar to the Asian countries Japan (0.23) and South Korea (0.21); and to Poland (0.22) [16, 39, 52].

For MPS II, Norway reported the lowest incidence rate in the literature (0.13), and Estonia the highest (2.16) [51, 53]. In the present study, a US incidence rate of 0.26 was reported, which is similar to some Scandinavian countries such as Sweden (0.27) and Denmark (0.27).

In regard to MPS III, the incidence ranges between 0.25 and 100,000 live births in South Korea and 1.89 in the Netherlands [52, 54]. In the US, the current study calculates MPS III incidence at 0.26. Although on the lower end of the spectrum, this figure is similar to that of South Korea as well as that of Norway (0.27) [39, 52].

Additionally, for MPS IV, a study from Sweden estimated the country’s incidence at 0.07 per 100,000 live births, while another Scandinavian country, Norway, estimated the highest incidence for this MPS type (0.76) [51]. This study calculates MPS IV incidence at birth in the US to be 0.14, which is again on the lower spectrum of this range.

For MPS VI, the incidence ranges between 0.013 and 100,000 live births in Poland to 0.29 in British Columbia [16, 55]. The incidence in the US for MPS VI was calculated to be 0.04, which is closer to the incidence estimated in Japan (0.03), Denmark (0.05), and the Czech Republic (0.05) [51].

For MPS VII, many studies did not calculate incidence, as this MPS subtype was not present in their patient population, although many papers attribute this to the early fatality of the disease and differing detection methods in each country. Based on the reported MPS VII cases, the incidence varies between 0.02 in Japan and the Czech Republic [39, 56], and 0.29 in British Columbia [55]. The present study calculated MPS VII incidence in the US to be 0.027 based on data provided by Ultragenyx Pharmaceuticals. Of note, per the National MPS Society’s registry, the incidence rate was found to be 0.008, most likely due to the database’s limitations as membership of the National MPS Society.

Another potential problem for the MPS patient population is the possibility of being misdiagnosed. Although definite figures regarding an estimate on MPS lifelong misdiagnosis cannot be accurately approximated, it is important to recognize that the multisystem involvement of MPS may have an initial presentation similar to other diseases [57]. Consequently, initial challenges in making this diagnosis include the fact that many specialists separately examine seemingly unconnected health problems. Hip disease, common in most MPS subtypes, could be mislabeled as Legg-Calve-Perthes (LCP) disease or spondyloepiphyseal dysplasia (SED), whereas developmental issues might be attributed to neurodevelopmental syndromes like ADD (attention deficit disorder). Additionally, the myriad of storage disorders that can mimic multiple of these symptoms (i.e. sphingolipidoses, mucolipidoses), along with the lack of newborn screening for MPS, and the lack of availability of POC tests in primary care offices, are additional factors that contribute to the misdiagnosis of these diseases.

We calculated the overall MPS prevalence to be 2.67 per million. This is almost half the prevalence for all MPS disorders in Scandinavian countries such as Sweden (4.24 per million), Norway (7.06) and Denmark (6.03) [51]. It is important to note that the US presents a larger and more diverse population than most countries, with over 4 million live births per year, which may explain the low incidence and prevalence yielded in this study. However, it is also key to acknowledge that this registry does not include the totality of MPS cases in the US. The lack of data from pharmaceutical companies, as well as children’s hospitals, might have contributed to an underestimation. Although a review of other countries also demonstrates heterogenous data, the smaller size of European countries and lack of state fragmentation makes it easier to estimate incidence and prevalence data for that geography. A recent report by Borges et al. estimated the prevalence of MPS using publicly available population based genomic data [58]. This approach overestimated MPS prevalence due to limitations of (1) the database, (2) filtering steps and consensus scores used in the methodology, and (3) lack of estimation of false positive results, among others [58].

In this study, the main source of data originated from the National MPS Society and Ultragenyx® Pharmaceuticals. However, National MPS Society data may not include all MPS patients within the US as patients are not required to be a member of the society. Additionally, it is possible that patients joined another MPS society focused on their specific subtype, such as the Morquio registry, instead of the National MPS society, to explain some of the lower incidence rates of MPS IVA.

This study began as a search for MPS incidence and prevalence rates in the US. However, as previously mentioned, this study also revealed that membership to the National MPS Society is not uniform throughout the US. The differences in newborn screening standards across states may also be an important factor for lower numbers, as MPS may not be included in the screening process at all. Some limitations of our study include: (1) lack of information from pharmaceutical companies that have patient registries and manufacture ERT for MPS disorders, (2) underestimation of the number of MPS patients based solely on those that join the National MPS Society, (3) lack of acceptance of the diagnosis by families and reluctance to join the membership, (4) lack of awareness of the National MPS Society’s existence, and (5) discrepancies in incidence rate by state could be explained by differences between patients registered versus patients born in a state. To accommodate for the US’ much larger and divided population, the authors propose that the best method of record keeping for rare diseases would be a national rare disease registry with mandated reporting from every state to ensure accurate epidemiological information.

## Conclusions

To date, this is the most comprehensive review of the incidence and prevalence rates of MPS in the US. Policy advocacy is needed to establish newborn screening of MPS in all states. Furthermore, a national registry for all rare diseases is needed to ensure accurate epidemiological information.

## Methods

### Study aim and design

The study aims to identify MPS incidence and prevalence in the US at national and state levels and to guide clinicians and policy makers utilizing a retrospective study design.

### Study population

The Institutional Review Board (IRB) at Saint Louis University determined that our human subjects research was exempt from a formal IRB submission due to lack of patient identifiers or protected health information (PHI). An attempt to use a database of over 200 Children’s Hospitals was unsuccessful. Due to the lack of consistent information and the reluctance of many hospitals to provide de-identified patient data, it was not possible to include cases from these hospitals. Additionally, clinical trials information on MPS studies was obtained (clinicaltrials.gov) but is excluded from the present study due to methodological issues including difficulty to assess if there were duplicated cases owing to many patients participating in more than one trial.

Another potential source of data is pharmaceutical companies that manufacture enzymes to treat MPS types including BioMarin® Pharmaceuticals (MPS I, IVA, and VI); Shire® Pharmaceuticals (MPS II); Ultragenyx® pharmaceuticals (MPS VII), and Genzyme® (MPS I). All were contacted for information on the number of MPS cases in the US. However, only Ultragenyx® Pharmaceuticals provided the overall number of cases for MPS VII reported until 2015. The best source of data was The National MPS Society (http://mpssociety.org/) who provided a de-identified database of all MPS members, which included all 50 states from 1995 to 2015. This period of time was used to calculate the incidence rate at birth and prevalence of MPS in the US. The database included year of birth, year of death, type of disease, and state of residence. The National MPS Society is an organization that advocates for MPS patients by providing social and medical support, championing newborn screening for MPS, funding MPS-focused research to find a cure, and streamlining the pathway from research to treatments (https://mpssociety.org/). Their free membership requires newly diagnosed patients or family to fill out a brief survey. Data from the National MPS Society are self-reported and collected as part of the membership registration. Membership provides members with access to MPS publications, physician databases, and eligibility for financial assistance grants and scholarships.

We included information of the number of patients with MPS IVA in the US from the published study of the International Registry for Morquio A Disease (1998–2006) [7]. Additionally, literature on MPS I newborn pilot studies from six states (Missouri, Illinois, Washington, North Carolina, Kentucky, and New York) was included, and their incidence figures were compared to the state-stratified data calculated in the present study.

### Data handling and analysis

All the information was compiled in a database used for statistical analyses. The National Center for Health Statistics data was used to obtain estimates on US population from the years 1995 to 2015. The incidence rate, also referred to as birth prevalence in the literature, was calculated by dividing the total number of cases by the total number of live births during the study period. Period prevalence was calculated by dividing the total number of cases between 1995 and 2015 by the average of the total population during the study period per 1 million people.

Incidence was sub-analyzed based on state location. The number of live births was obtained for each state using the National Center for Health Statistics data between 1995 and 2015. Additionally, data from newborn screening pilot studies in multiple states were included and their respective incidence rates were reported and compared to the rates yielded by the current study. Geographic Information Systems (GIS) was used to map state-level incidence rates in the country.

## Data Availability

The datasets used and/or analyzed during the current study are available from the corresponding author on reasonable request.

## Abbreviations

ACHDNC: Advisory Committee on Heritable Disorders in Newborns and Children
ARSB: *N*-Acetylgalactosamine-4-sulfatase
CS: Chondroitin sulfate
DS: Dermatan sulfate
ERT: Enzyme replacement therapy
GAGs: Glycosaminoglycans
GALNS: *N*-Acetylgalactosamine-6-sulfate sulfatase
GIS: Geographic information systems
GLB1: β-galactosidase
GNS: *N*-Acetylglucosamine 6-sulfatase
GUS: β-d-glucuronidase
HGSNAT: α-Glucosaminidase acetyltransferase
HS: Heparan sulfate
I2S: Iduronate-2-sulfatase
IDUA: α-l-Iduronidase enzyme
KS: Keratan sulfate
MPS: Mucopolysaccharidoses
NAGLU: α-*N*-Acetylglucosaminidase
SGSH: Heparan-*N*-sulfatase
US: United States
